# An evaluation of casein hydrolyzate in combination with antibiotic for bacterial cure and subsequent increase in milk yield in dairy cows

**DOI:** 10.1186/1746-6148-7-3

**Published:** 2011-01-07

**Authors:** Gabriel Leitner, Shamay Jacoby, Nissim Silanikove

**Affiliations:** 1 National Mastitis Reference Center, Kimron Veterinary Institute, P.O.B. 12, Bet Dagan 50250, Israel; 2 Biology of Lactation Laboratory, Institute of Animal Science, the Volcani Center, P.O.B. 6, Bet Dagan 50250, Israel

## Abstract

**Background:**

A 3-yr study examined whether prepartum treatment with casein hydrolyzate in combination with antibiotic, as routinely used in Israel for dry cow therapy, improved bacterial cure and increased milk yield in subsequent lactations in comparison with treatment with antibiotic alone. The vast majority of bacterial isolates in samples collected prior to drying-off comprised coagulase-negative staphylococci, mostly as *Staph. chromogenes*.

**Results:**

Bacterial cure associated with the combined treatment was 73.8% in cows, significantly higher than the 51.7% cure recorded when cows were treated only with antibiotic. During the study, the annual milk yield of non-casein hydrolyzate treated and treated control cows increased at ~2% per year, which is consistent with the national annual increase attributed to genetic selection. In cows treated with casein hydrolyzate the increase was 9% (above the 2% expected) in the first lactation after the treatment, and 6.3% (above the 4% expected for 2 years) in the second lactation after treatment. These increases were significantly higher than those in the controls and those expected through genetic improvement.

**Conclusions:**

Treatment with casein hydrolyzate at dry-off was shown to be a viable mean to eliminate existing environmental bacterial infection, and to improve milk yield in the next lactation.

## Background

Active involution of the mammary gland is induced by milk stasis, which in turn triggers a negative feedback regulatory mechanism that leads to precipitous dry off of milk secretion and shrinkage of the secretory tissue (parenchyma) of the mammary gland [1,2]. Involution in cows [3-6] and mice [7,8] is associated with an inflammatory response that is characterized by activation of the glandular immune system, accelerated apoptosis of epithelial cells, and tissue remodeling [1,2,9]. The process of active involution that follows milk stasis in goats [10] and cows [11] can be triggered by the induction of intensive plasmin activity in milk; this, in turn liberates casein-derived active peptides [12]. Infusion of casein hydrolyzate (CNH), which contains active casein-derived peptides, dramatically accelerated the involution, which was completed within 3 days in goats and cows, compared with 3-4 weeks without such treatment, and resulted in a marked reduction in milk yield (MY) even after the first day of treatment [10,11,13]. These responses were manifested in the mammary secretion within 8 h after the first application, which relates to the forceful activation of the innate immune system and the drastic reduction in nutrient availability (mainly lactose) for bacterial growth [13].

These properties of CNH were exploited successfully to eliminate quarter-udder infection by drying-off the infected glands during lactation, which resumed normal milk secretion in the subsequent lactation [14]. Moreover, it was found that infusion of CNH induced marked bactericidal and bacteriostatic responses against udder pathogens, such as *Escherichia coli *and *Staphylococcus aureus*, in the involuted gland secretion as tested *in vitro *[13].

Casein hydrolyzate may be used as a tool to reduce the discomfort associated with drying-off of modern high-yielding dairy cows, by preventing the enormous udder engorgement that typically develops with this management process [15].

It has been well established that the beginning of the dry period in dairy cows is associated with an increased risk of acquiring intramammary infection (IMI)[16]. This increased risk is closely related to MY at drying-off, and may be related to increased incidence of milk leakage from the udder [17] and the slow activation of the innate immune system [13]. Each 5 kg/d increase in MY above 12.5 kg/d at dry-off increase the probability of acquiring new IMI with environmental bacteria by 77% [17]. Thus, in Israel and in other countries, most cows are at high risk of acquiring IMI at drying-off, because they are still producing milk at over 20 L/d.

It was hypothesized that acceleration of the involution process might increase bacterial cure [18-20] and result in increased MY in the subsequent lactation [1]. The aims of the present study were to determine whether intramammary CNH treatment at drying-off would result in improved bacterial cure of existing IMI, and increased MY in the subsequent lactation. Since it was the first time that the efficacy of CNH as dry cow treatment (DCT) was tested and because the study was performed in a commercial herd, a combination of CNH with an antibiotic treatment was used instead of CNH alone. Due to management limitations, CNH was tested only during one year of the 3-year study. For testing the efficiency of the CNH treatment for curing/preventing bacterial infection the year prior to the CNH usage and the year after served as control. To overcome the effects associated with genetic improvement, the MY of heifers, which entered the herd throughout the 3-year study was analyzed and served as a covariate.

## Methods

### Materials

A commercial nafcillin/procaine penicillin G/dihydrostreptomycin preparation, Nafpenzal DC (Intervet, the Netherlands), which is a medication for DCT, was used according to the manufacturer's instructions. A pre-commercial prototype of the CNH was prepared under Good Laboratory Practice conditions [15] and was used in combination with Nafpenzal DC for DCT. The CNH solution was a sterile pyrogen-free liquid preparation that was stored in sterile vials [15]. Each infusion into a quarter contained 10 mL of CNH, with a peptide concentration of 7.2 mg/mL followed by a Nafpenzal DC tube.

### Study design

All protocols were approved by the Institutional Animal Care Committee of the Agricultural Research Organization, which is the legitimate body for such authorizations in Israel.

The study extended over 3 years and is summarized in Table 1; it covered Israeli Holstein cows (pluriparous; second lactation and above) and heifers (first lactation) of the commercial-experimental herd of the Agricultural Research Organization, Bet Dagan, Israel, which holds 250 animals. Cows and heifers were held in high-roof barns suitable for sub-tropical conditions, and were fed a typical Israeli TMR comprising 65% concentrates and 17% protein. The food and water managers were located in the shed. During the summer months (June to September) the cows are routinely cooled by fans and sprinklers. Two dry-off treatments were compared: Nafpenzal DC, and Nafpenzal DC combined with CNH infused to each quarter in the treated cows. The cows were treated in accordance with their dry-off schedules. Cows included in "Period 1" (August 2004 to August 2005) were dry-off treated with antibiotic. In the following season, from September 2005 to June 2006 (Period 2), the vast majority of the cows in the herd were dry-off treated with a combination of antibiotic and CNH, and a group of 17 cows, that served as control cows, were treated only with antibiotic for DCT. In the third season of observation, from July 2006 to April 2007 (Period 3), all the cows were dry-off treated with antibiotic. All the heifers that joined the herd during Periods 1 to 3 were included to enable comparison of the evolution of their MY with that of the experimental cows. Heifers were not treated for DCT. The number of animals recorded in each season is indicated in Table 1. Because of the natural dynamics of the herd population, 41 cows were observed during all 3 Periods, 101 cows in 2 Periods and 162 cows in one Period; a total of 481 cows (Table 1).

**Table 1:** Experimental structure: animals (number and types), treatments, and Periods

*Period*	*Time*	*Animals*	*Number*	*Treatment*
1	Aug. 04 - Aug. 05	heifers	74	None
		cows	111	Naf. DC
2	Sep. 05 - June 06	heifers	56	None
		cows	92	Naf. DC + CNH
		control cows	17	Naf. DC
3	July 06 - April 07	heifers	48	None
		cows	83	Naf. DC

Naf. - Nafpenzal

The average MY in this farm at 2005 was 11,135 L during 305 days of lactation and the average bulk tank somatic cell count (SCC) was 236,000. Milk yield and SCC were retrieved from the routine monthly record of this information for each Holstein cow in Israel by the Israeli Cattle Breeders' Association. All animals were sampled by quarter, for SCC and bacterial udder infection, as follows: 3 times at weekly intervals during the last month before drying-off; at the time of parturition (days 0 to 3); and monthly for up to 100 (range 95 to 105) days during the subsequent lactation. Thus, each cow was sampled 4 times during this period. A cow was considered infected when at least two of the milk samples were positive with the same bacterium accompanied by increased SCC.

### Bacteriological analysis

Duplicate quarter foremilk samples were taken aseptically and submitted to the laboratory within one h. Bacteriological analysis was performed according to accepted standards of microbiological procedures as described by the National Mastitis Council [21]. All samples were taken during milking. Udder quarters were cleaned and disinfected prior to sampling with a non-woven disposable commercial wipe that was moistened with chlorhexidine, cetrimide and ethanol. The three first squirts of milk were discarded and approximately 3-5 ml of milk were taken in sterile tubes. From every milk sample, 0.01 mL was spread onto blood-agar plates (Bacto-Agar; Difco Laboratory Becton, Dickinson and Company, France) containing 5% of washed red sheep blood cells and on MacConkey plates. All plates were incubated at 37°C and examined for growth after 18 and 42 h. If there was no agreement between duplicates the quarters were sampled again. Colonies suspected to be staphylococci were tested for coagulase (tube test, Anilab, Rehovot, Israel). Species identification was carried out with the API STAPH-IDENT, 32 Staph kit or rapid ID 32 STREP (bioMerieux S.A., 69280 Marcy l'Etoile, France). When micrococci-like bacteria in given sample matched the reference kit specie by > 90%, the specie was regarded as specific.

Bacterial cure was defined as the absence of the bacteria identified in the same quarter in the month preceding drying-off from the milk sampled during the first 100 days of a new lactation (i.e., in at least 4 postpartum milk sampling). New infection was defined as the appearance of infection that was absent from samples taken before drying-off in the previous lactation in a given quarter at the start of the new lactation. In order to be defined as new infection, the infection could be identified in milk sampled during the first 3 d postpartum, or in the first month of postpartum samples;, it should have persisted for at least 2 additional samplings, i.e., during the first 100 days of lactation. Clinical infections were defined by the herd veterinarian by for-stripping milk of suspicious cows. The SCC in milk was determined in the central laboratory of the Israeli Dairy Cow Breeders' Association, according to established standard procedures [22].

### Statistical Analysis

All the statistical analysis procedures were carried out with the JMP software [23]. All the main measures (cure rate, CC and milk yield) were analyzed according to the following model:

$${\text{Y}}_{\text{ijk}}=\mu +\alpha_{\text{i}}+\beta_{\text{j}}+\alpha \beta_{\text{ij}}+{Κ}_{\text{l}}+\alpha {Κ}_{\text{il}}+{\text{e}}_{\text{ijkl}}$$

where μ = Mean of all data, α_i _= effect of Period, i (i = 1, 2 or 3); βj = effect of cow category (j = 1, 2 or 3 for heifer, experimental, and control cow, respectively); αβ_ij _= the cow category × Period interaction; Κ_l _= effect of lactation number of cows (l = 2, 3, or > 4); αΚ_il _- interaction of period and cows lactation number and e_ijkl _= residual variance between measurements (random error).

Because the effects of Κ_l _and αΚ_il _were not significant, similar statistical results were obtained when treatments were also analyzed by the Pearson's chi-square (χ^2^) test: Nafpenzal DC and Nafpenzal DC combined with CNH were compared by the χ^2 ^testfor their effects on: quarter's bacterial cure and acquisition of new infections in experimental cows from Periods 2 and 3. For simplicity, only the χ^2 ^analysis was presented in Table 2.

**Table 2:** Bacterial status (infected, noninfected, subclinical, clinical and cured) in Period 2 (experimental, following Nafpenzal DC + CNH at DCT) and Period 3 (control, following Nafpenzal DC)

		*Period 2 *(experimental period)	*Period 3 *(control period)	*P [χ^2^]*
Before drying-off	Cow/glands	92/368	83/332	NS
	Uninfected	326/368 (88.6)	274/332 (82.5)	NS
	Infected	42/368 (11.4)	58/332 (17.5)	NS
Postpartum	Uninfected^1^	312/326 (95.7)	259/274 (94.5)	NS
	Subclinical^2^	14/326 (4.3)	15/274 (5.5)	NS
	Clinical^3^	28/326 (8.6)	20/274 (7.3)	NS
	Cured^4^	31/42 (73.8)	30/58 (51.7)	0.025
	Not Cured ^5^	11/42 (26.2)	28/58 (48.3)	0.025

^1 ^Uninfected quarter before drying-off and uninfected at parturition.

^2 ^New Infection, subclinical - Infection was detected during parturition and in the first 100 days in the new lactation: The same udders were uninfected before drying-off.

^3 ^New Infection, clinical - Infection was detected during the first month after parturition and remained for the first 100 days in the new lactation: The same udders were uninfected before drying-off.

^4^Cure - Bacteria detected in the month preceding dry-off was not detected in the same udder during the first 100 days of lactation

^5 ^Not Cured - The reciprocal of cured: Bacteria detected in given udders in the month preceding dry-off was also detected in the same udder during the first 100 days of the subsequent lactation.

Milk yield comparisons were based on the monthly MY during the first 5 months of lactation according to the above model.

The above statistical model with the inclusion of the effect of SCC (categories of < 50,000; 50,000 to 700,000; and > 700,000) and the interaction of SCC with period effect was used to analyze the effect of SCC on milk yield. Alternatively, for all three periods, the effect of the same SCC categories on milk yield was analyzed by the χ^2 ^test. The statistical analysis by both methods, yielded similar predictions, and for simplicity the χ^2 ^test results are presented in Table 3.

**Table 3:** Distribution of SCC (cells/ml) in Periods 1, 2 and 3.

*SCC (category)*		*Period*		*P[χ^2^]*
	1	2	3	
< 50,000	54.9	65.7	66.3	NS
50 - 700,000	24.4	15.8	15.7	NS
> 700,000	9.9	9.2	10.1	NS
Clinical infection	10.8	9.3	7.8	NS

## Results

### Bacteriology

Of the 92 cows (368 glands) that gave birth during Period 2 and had been treated with Nafpenzal DC in combination with CNH in the dry period at the end of Period 1, 88.6% of the glands (326/368) were uninfected, and infection was detected in 11.4% (42/368) at dry-off (Table 2). Of the 326 uninfected glands, 312 (95.7%) remained uninfected through 100 d of the lactation (range 95 to 105 d), and 14 (4.3%) exhibited new bacterial infection immediately postpartum (day 0 to 3), and this persisted in sub-clinical forms through 100 d of the lactation. In 28 (8.6%) out of 326 glands newly acquired bacterial clinical infection was detected during the first month. Bacterial cure of cows entering the dry period infected was detected in 31 out of 42 glands (73.8%), and 11 out of 42 (26.2%) glands remained infected with the same bacteria (Table 2).

For comparison among the 83 cows (332 quarters) that gave birth during Period 3 and had been treated with Nafpenzal DC during the preceding dry period, 82.5% of the glands were bacteria free and 17.5% were infected at dry-off (Table 2). Of the 274 uninfected glands, 259, i.e., 94.5%, remained uninfected through 100 d of the lactation, and 15, i.e., 5.5%, exhibited new bacterial infection immediately postpartum (days 0 to 3), and this infection persisted sub-clinically through 100 d of the lactation. In 20 (7.3%) of the 274 glands, newly acquired clinical bacterial infection was detected during the first month of lactation (Table 2). Bacterial cure of cows that entered the dry period infected was observed in 30 out of the 58 glands (51.7%), and 28 (48.3%) glands remained infected with the same bacteria (Table 2).

The vast majority of bacterial isolates (92%) in the month preceding the dry period were identified as coagulase negative staphylococci (CNS), mostly *Staph. chromogenes *(85%). Thus, 'bacterial cure' by DCT in the present study refers almost exclusively to cure from CNS infection. New infections that were identified on day 0 were not associated with clinical mastitis and were caused exclusively by CNS (7%); these infections developed into chronic infections that persisted for at least 100 d. The vast majority (93%) of bacterial infections detected during the first month caused clinical mastitis; *E. coli *and *Streptococcus dysgalactiae *were identified as the causative agents. Based on their absence in the next samplings, it can be concluded that these infections were cured either spontaneously (mostly *E. coli*) or by treatment with antibiotic. In few cases (1 to 2 per lactation), the infection did not cured and was a ground for culling. Thus, routine antibiotic treatment with Nafpenzal DC at dry-off provided only 51.7% cure whereas this treatment in combination with CNH significantly increased the cure to 73.8% (Table 2*P *= 0.025). No differences were found between the treatments in new infection rates, either with CNS during the dry period or with *E. coli *and *Streptococcus dysgalactiae *during the lactation.

### Somatic Cells

The proportion of udders with SCC less than 50,000 cells/mL increased from 54.9% in Period 1 to 65.7% in Period 2 and to 66.3% in Period 3, whereas the proportions with SCC in the range of 50,000 to 700,000 were 24.4, 15.8, and 15.7% in Periods 1, 2, and 3, respectively. Although the proportion of cows with lower SCC increased in Periods 2 and 3, the differences were not significant (Table 3). The numerical changes in the rates of SCC > 700,000 and of clinical infections in the 3 Periods were not significant.

### Milk Yield

**Figure 1:**
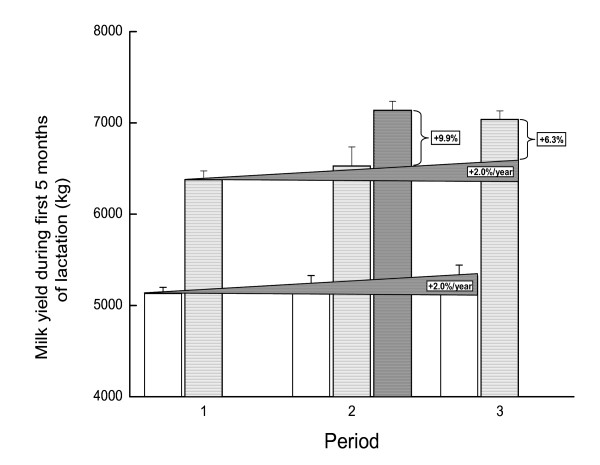
**Milk yield during first 5 months of lactation (kg)**.

The MY of the heifers increased by 2% (about 220 L per cow) each year, from 5,130 L over 5 months in Period 1 to 5,323 L over 5 months in Period 3 (Figure 1). A similar increase of about 2% from Period 1 to Period 2 (6,374 to 6,526 L over 5 months) was recorded in the 17 control cows. The increase in MY among the heifers and the pluriparous control cows was not significant. In contrast, a significant (*P *< 0.0001) increase in MY from Period 1 to Period 2 by about 760 L per cow (i.e., from 6,374 in the first 5 month of period 1 to 7,132 L per cow in the first 5 month of period 2 following CNH treatment at dry-off of period 1) over 5 months was recorded in the pluriparous cows. Thus, the yield increase of the tested cows was 9.9% higher than the expected 2% per year (*P *< 0.001). These significant difference (*P *< 0.0001) were maintained in Period 3, in which the increase in MY was 10.4% higher than in Period 1 (from 6,374 in the first 5 month of period 1 to 7,033 L in the first 5 month of period 3 following CNH treatment at dry-off of period 1), (6.3% higher the 4% expected; *P *< 0.001). These differences remained highly significant, even after accounting for the natural annual 2% increase in MY in the whole herd, as recorded in the control groups of heifers and cows (Figure 1).

Analysis of the effect of SCC on MY (Table 3), indicates that the non-significant reduction in SCC level in the experimental cows cannot explain the increase in MY in those cows.

## Discussion

### Study Design

The present study was a feasibility study intended to analyze the effect of CNH treatment in a commercial herd with minimal interference to its day-to-day routine, which explains the somewhat unusual experimental design. The potential pitfalls in such a design are that it is subjected to confounding results because of year-to-year variations of food composition, environmental conditions, management etc. Thus, a special precautions in the experimental design included the use of heifers as control for changes relating to genetic improvement of MY, to account for such interferences, and therefore the results reflect the studied effects of the CNH dry-off treatment.

### CNH Treatment Improved Bacterial Cure of CNS

The present results show that treatment with antibiotic plus CNH DCT was effective in curing CNS IMI. This is important because: (i) it provides a means for preventing deterioration in milk quality and associated economic losses; and (ii) it provides a potential means to prevent the hazard associated with routine use of high doses of antibiotic, with blanket DCT procedures, that have become the accepted tool to control IMI in Israel and other countries. Although in the present study, treatment with CNH was accompany by antibiotics, the significant improvement in bacterial cure in comparison to treatment with antibiotic alone under farming conditions provided evidence that intra-mammary CNH treatment has the potential for being developed into an effective non-antibiotic, non-hormonal DCT for bacterial cure. Nevertheless, in order to advance this potential, the benefit in using CNH over conventional antibiotic DCT should be demonstrated in a controlled study at dry-off.

A decade ago, CNS were often regarded as pathogens of minor importance [24,25], especially in comparison with *Staph. aureus*, streptococci, and coliforms, which may cause severe mastitis. Nevertheless, in many countries, including Israel, CNS are the predominant pathogens associated with mastitis [24-27]. Mastitis caused by CNS usually remains subclinical or mildly clinical [28,29], but it may decrease milk production [22,30]. Leitner et al. [22,31,32] have shown that losses in curd yield caused by the use of CNS-infected milk are several times as great as the milk losses, because of simultaneous deterioration in milk quality. Thus, if milk is being allocated for cheese production, the economic burden imposed on dairies by loss in curd yield is greater than that imposed on farmers by loss of MY [22].

Although we cannot exclude the possibility of reinfection with the same species following its elimination, we assume that in the present study the CNS infections recorded immediately postpartum persisted in the udder through the entire lactation. This conclusion is consistent with evidence that CNS are capable of persisting in the udder for months or even throughout the lactation [28,29,33].

Some studies have found rather high spontaneous CNS cure rates of about 60 to 70% during the dry period [34], whereas others found markedly lower rates, ranging from 15.5 to 44% [16,35]. In a broad survey of heifers in the USA and Canada it was found that quarters that were infected prepartum, mostly with CNS, and were treated with antibiotics had a 59.5% cure rate, compared with a spontaneous cure rate of 31.7% [36]. A study on heifers in 2 research dairy herds yielded a very similar CNS cure rate [37]. The somewhat lower cure rate obtained with antibiotic DCT in the present study may be related to the fact that our subjects were cows, or to the stricter criteria we applied to define a cure, compared with that used in standard definitions, i.e., absence of infection during the first month of lactation. Burriel [38] found that CNS can adhere to and produce slime in various tissues, including epithelial cells of the mammary gland. Thus, the absence of CNS from a culture of a given sample does not necessary indicate their absence from the sampled tissue [39]. This hypothesis is supported by the findings of a large study in Norway that covered a total of 684 cows from 288 different herds in 3 Norwegian regions, DCT of infected quarters led to a 5.2:1 greater probability of the quarters being healthy within 30 ± 17 days into the next lactation than that in control (non-treated) groups. Nevertheless, the overall frequency of CNS in the sampled quarters of cured cows at the end of lactation was no different from that among of the control cows [40]. The above findings justify our more strict criteria for defining a cure, and also emphasize that the success of the present CNH treatment in significantly improving the CNS cure rate was genuine.

In the present study none of the cows were infected with *Staph. aureus*, and the numbers of cows infected with coliforms and streptococci were too low to assess the efficacy of the CNH treatment in curing or preventing such infections. However, in a previous study, CNH treatment of single quarters during lactation has been shown to be effective in curing chronic and subclinical mastitis associated with these bacteria [14]. Thus, it is worth further investigating the potential benefit of applying this method as a DCT to cure and reduce the SCC counts in herds having higher than the typical proportion of coliforms, streptococci and *Staph. aureus *IMI at dry-off in Israel.

### DCT with CNH Increased Milk Yield

The progressive increase in MY at 2% per year in heifers or in the control cows is typical of the yearly improvement in MY attributed to genetic improvement in Israeli dairy herds [41]. Thus, the increase in MY that was recorded in the experimental cows considerably exceeded any plausible trend. No such response was recorded in cows treated with antibiotic only, i.e., the 17 control cows and those observed in Period 2 that had joined the trial as heifers in Period 1 and 2, therefore this MY enhancement can be related exclusively to the effect of treating the cows with CNH.

Two hypotheses may account for this increase. First, it may relate to the higher cure rate and the reduction in the population of SCC that typically is negatively related to MY [20]. However, analysis of MY as related to SCC distribution indicates that in the present case the effect of CNH treatment on lowering of SCC was far from significant, probably because the infection rate and, therefore, the SCC, were initially low and also because treatment did not affect new IMI in the subsequent lactation. Thus, in herds with poorer initial conditions, this effect would be expected to be more noticeable. The second hypothesis relates to the fact that treatment with CNH accelerated gland involution [11,13]. Thus, this procedure probably increased epithelial cell apoptosis at the start of the dry period. As the gland parenchyma rebuilt itself during the period preceding the subsequent parturition, it may be expected that it would be composed of larger proportion of newly synthesized epithelial cells, which would be expected to enhance the gland milk production capacity [1]. Our results suggest that this effect persisted through two subsequent lactations.

## Conclusion

This study provided evidence that intra-mammary CNH treatment has the potential for development as an effective non-antibiotic, non-hormonal DCT for bacterial cure and for enhanced MY in the subsequent lactation. Further research along this line is therefore warranted, particularly under the following conditions: (i) In herds presenting broader bacterial infection status and higher bulk tank SCC; (iii) In combination with teat sealers, thus contributing also to prevention of new IMI around parturition (iv) In exploiting the accelerating effect of CNH treatment on the involution rate, to shorten the dry period without impairing the next-lactation MY, or even improving it and (v) Because it is a natural product, CNH treatment has the potential to prevent the transmission of methicillin resistant genes and those resistant to other antibiotics.

## Authors' contributions

GL and NS were responsible for performing the experiments and for the analysis of the data, interpretation of the results and the writing of the manuscript. NS prepared the CNH solution and wrote the first draft of the paper. SJ is the farm manager and participated in performing the experimental treatments. All authors read and approved the final manuscript.
